# Quality of Life and Predictive Factors among Patients with an Abdominal Stoma: A Cross-Sectional Study in a Teaching Tertiary Hospital

**DOI:** 10.21315/mjms-12-2025-924

**Published:** 2026-04-30

**Authors:** Patimah Abdul Wahab, Nurhazelin Mohamed, Norsuzana Ismail, Siti Zuhaidah Shahadan, Intan Idiana Hassan, Haryanto Haryanto

**Affiliations:** 1 Department of Medical Surgical Nursing, Kulliyyah of Nursing, International Islamic University Malaysia, Kuantan, Pahang, Malaysia; 2 Operating Theatre, National Heart Institute, Kuala Lumpur, Malaysia; 3 Department of Nursing, Sultan Ahmad Shah Medical Centre @IIUM, Kuantan, Pahang, Malaysia; 4 Department of Nursing, School of Health Sciences, Universiti Sains Malaysia, Health Campus, Kota Bharu, Kelantan, Malaysia; 5 Department of Wound Care and Critical Care, Institut Teknologi dan Kesehatan Muhammadiyah Kalimantan Barat, Pontianak, Indonesia

**Keywords:** quality of life, ostomy, surgical stomas, colonic neoplasms, Malaysia

## Abstract

**Background:**

An abdominal stoma is instrumental in preserving life; however, its impact on a patient’s quality of life (QoL) is multifaceted and influenced by a complex interplay of various factors, including sociodemographic and clinical factors. This study aimed to assess patients’ QoL and examine its relationship with these factors.

**Methods:**

A cross-sectional telephone survey was conducted among patients with abdominal stoma at a teaching tertiary hospital from December 2020 to February 2021. Participants were identified from the medical records of postoperative patients with a stoma and underwent eligibility assessment via medical record reviews. Data on sociodemographic characteristics and clinical conditions were obtained from medical records. A telephone survey employing the City of Hope-Quality of Life-Ostomy Questionnaire (CoH-QoL-OQ) facilitated QoL data collection. Predictor factors of QoL were examined using multiple linear regression.

**Results:**

The mean QoL score was 6.86 (standard deviation [SD] = 0.84), indicating a moderate QoL. Physical well-being was the most affected (6.29; SD = 0.76), and spiritual well-being was the least affected (8.07; SD = 1.17). Employment status (*b* = −0.94 [95% CI: −1.67, −0.22]; *P* = 0.014) and colorectal cancer (CRC) (*b* = 0.92 [95% CI: 0.13, 1.71]; *P* = 0.025) were significant predictors of QoL (*R*^2^ = 0.389).

**Conclusion:**

The study found that patients with stomas generally experience a moderate QoL, with poorer QoL associated with unemployment and non-CRC diagnoses. These findings provide valuable insight into the need to strengthen patient-centred care to ensure appropriate and equitable supportive interventions for all patients.

## Introduction

An abdominal stoma is a surgical opening on the surface of the abdomen through which a small or large intestine or urinary tract is connected to the external body. This intervention becomes paramount in preserving a patient’s life, offering an alternative pathway for waste elimination when the typical digestive or urinary systems are compromised. The two primary types of abdominal stomas are colostomy and ileostomy, which serve the digestive system, as well as urostomy for the urinary system. Colostomy is the most common type, often linked to colorectal cancer (CRC), a widespread and life-changing disease affecting many around the world (1). Globally, CRC ranks as the third most common cancer and the second-leading cause of cancer-related deaths (2). In Malaysia, CRC stands as the second most prevalent cancer for both men and women, accentuating its considerable impact (3). The increasing global incidence of CRC is expected to fuel the growing prevalence of ostomies. Apart from CRC, some factors, such as trauma, intestinal obstruction, ischaemia, or inflammatory bowel diseases (IBD), also necessitate the diversion of faeces or urine.

Worldwide, studies showed that living with an abdominal stoma can be challenging and significantly impact the quality of life (QoL) (4–7). A study in Europe to determine the factors associated with decreased QoL in the long-term postoperative period after surgical treatment of CRC found that stoma formation is a predictor of worse QoL (6). Meanwhile, a study examining the QoL in rectal cancer patients in China who underwent either non-ostomy or ostomy surgery found that patients undergoing ostomy surgery, particularly those with low and super-low rectal cancer, experienced poorer QoL (8). In Malaysia, research on patients with abdominal stoma is limited (9). One study conducted among Malaysian patients diagnosed with CRC who underwent ostomy surgery also highlighted a notable decline in their QoL compared to those who did not undergo the procedure (5). More research is needed to understand the QoL outcomes and to develop targeted interventions to support these patients.

The QoL of individuals with an abdominal stoma was reported to vary in range (1, 10). Coping with a stoma presents both physical and emotional challenges that can interrupt daily activities and contribute to a diminished QoL. The adjustment to life with a stoma often triggers emotional distress, particularly in newly operated individuals (11). The detrimental effects of QoL include sexual concerns, depression, constipation, dissatisfaction with appearance, travel difficulties, fatigue, and noise worries (12). Additionally, studies indicate that a stoma negatively impacts not only the individual’s QoL but also their spouses (13). While an enhanced QoL is anticipated following stoma adaptation, it may also signify the culmination of holistic treatment and postoperative rehabilitation progress (10). Considering the multidimensional nature of QoL, it is crucial to assess various aspects of ostomy patients comprehensively.

The QoL for patients with abdominal stomas is influenced by several factors, including marital status, education level, the type of stoma, and the presence of stoma-related complications. Additionally, self-care capabilities and the knowledge and skills required for stoma management play crucial roles. Common issues such as pouch leakage and the risk of hernias significantly contribute to a diminished QoL for these patients (10, 12, 14, 15). Previous studies also show that older age, strong family support, social networks, effective communication with healthcare providers, spirituality, financial stability, and engagement in physical activity are associated with better QoL (13–16). Given the risk of poor QoL in this population, evaluating QoL and identifying key determinants is important for guiding supportive interventions and improving overall well-being. This study, therefore, aimed to evaluate the QoL of patients with abdominal stomas and assess the influence of sociodemographic and clinical factors.

## Methods

A cross-sectional telephone survey was conducted among patients with abdominal stomas from December 2020 to February 2021. Convenience sampling was used to recruit the participants of this study. The electronic health records of postoperative patients with stoma registered at a teaching hospital on the east coast of Peninsular Malaysia from November 2018 to February 2021 were retrieved and reviewed. Initial recruitment was conducted using several sources: the list of patients referred to the stoma nurse, admission records from the surgical ward, and patient registration records for follow-up treatment at the surgical outpatient clinic. Patients with abdominal stoma were identified, resulting in 48 patients being selected (9).

The eligibility of participants was subject to further screening prior to their invitation to engage in the study. The inclusion criteria specified that eligible individuals must be at least 18 years of age, possess abdominal stomas classified as colostomy, ileostomy, or urostomy, be proficient in either Malay or English, and voluntarily consent to participate in the study. The participants were excluded if their contact number was not available, if they had a significant disability such as memory impairment or deafness, or if they were deceased. The researcher proactively contacted patients via WhatsApp before the interview to briefly inform them about the study, outline the necessity of conducting a telephone interview for data collection, and encourage voluntary participation in the research.

If participants expressed interest, direct telephone contact was initiated to provide study details, obtain informed consent, and assure the confidentiality of their information. The informed consent form was shared via the phone application for participants to sign and return in the same manner. In instances where individuals faced challenges, a recorded verbal confirmation of their voluntary consent was deemed acceptable. For patients who did not provide a response or who lacked the requisite application, direct telephone outreach was conducted. Reasons for refusal to participate were documented. In this study, the estimated sample size was determined using the PS Power and Sample Size Calculation software, version 3.1.6 (Vanderbilt University, Tennessee, United States of America). A prior study reported a normally distributed response with a standard deviation (SD) of 1.46 (1). With a mean difference of 0.73, α = 0.05, and 80% power, the estimated sample size was 128.

### Instrument

The independent variables of this study comprised sociodemographic and clinical data, while the dependent variable is the patient’s QoL. Data collection was conducted using a structured questionnaire divided into three sections: sociodemographic information (including gender, age, educational level, marital status, and employment status), clinical information (encompassing type and characteristics of the stoma, CRC-related diagnosis, duration of stoma, and occurrence of complications), and QoL. The City of Hope-Quality of Life-Ostomy Questionnaire (CoH-QoL-OQ) was used to evaluate the patient’s QoL (17). This self-report instrument was selected because it comprehensively evaluates the multidimensional QoL of patients with a stoma and is among the most used tools for assessing the QoL of patients with an abdominal stoma (13, 18–20).

The CoH-QoL-OQ contains 43 questions, which can be divided into four domains: physical well-being (11 questions), psychological well-being (13 questions), social well-being (12 questions), and spiritual well-being (7 questions). The QoL score was rated based on a 10-point Likert scale ranging from 0 (worst outcome) to 10 (best outcome). Items 1 to 12, 15, 18, 19, 22 to 30, 32 to 34, and 37 were reverse-coded. The scores for each domain were computed by summing the scores of the individual items and then dividing the total by the number of items within that specific domain. This methodology was consistently applied to derive the overall QoL score. The total score ranged between 0 and 10, with 0 to 3 indicating low QoL, 4–7 for moderate QoL, and 8–10 indicating high QoL.

The instrument was tested in a previous study and was proven valid and reliable for measuring the QoL of patients with a stoma (17). In this study, the CoH-QoL-OQ was translated into Malay with permission granted by the original developer (17). A forward and backward translation process was performed, and discrepancies were reviewed and reconciled. The clarity and relevance of the translated items were ensured through a pilot study with five patients. Given the small sample size (*n* = 28), the researchers only managed to properly check for the content and face validity for use in the selected population. Based on the same sample size, the reliability of the instrument was checked, and the Cronbach’s alpha for the instrument and its domains ranged from 0.72 to 0.87.

### Data Collection

Data on sociodemographic and clinical characteristics were collected from the patients’ medical records. The data were verified and confirmed, missing data were completed, and QoL data were obtained from patients through a structured telephone interview. Figure 1 illustrates the recruitment flowchart of the participants and the data collection process in this study.

### Statistical Analyses

The data were analysed using Statistical Package for the Social Sciences (SPSS) version 29 (IBM Corp., Armonk, NY, US). Descriptive statistics, including frequency, percentage, mean, and SD, were used to present sociodemographic and clinical characteristics. Normality testing using the Shapiro–Wilk test showed a *P*-value greater than 0.05, which indicated that the data were normally distributed (21). Predictor factors of QoL were examined using multiple linear regression with the forward method to select variables. Independent variables with *P* ≤ 0.05 and significant clinical variables were included. Interactions and multicollinearity were checked, with no interaction if *P* > 0.05 and no multicollinearity if Variance Inflation Factor (VIF) < 10. A significance value of *P* < 0.05 was used for the analyses.

## Results

### Background of the Participants

This study included 23 participants, yielding a response rate of 18.0%. Among the 48 patients whose medical records were retrieved, 15 were deceased, and 10 did not respond to the call or had an unavailable telephone number. The mean age of the participants was 58.7 years (SD = 16.4). Overall, 69.6% of the participants were men (*n* = 16), and 30.4% were women (*n* = 7). The majority of the participants were Malays and Muslims (95.7%; *n* = 2), with Chinese participants comprising the remaining 4.3% (*n* = 1). The percentage of participants who underwent ileostomy was 56.5% (*n* = 13), while the percentage of participants who underwent colostomy was 43.5% (*n* = 10). Most participants (82.6%; *n* = 19) had temporary stomas, whereas 17.4% (*n* = 4) had permanent stomas.

### QoL of Patients with Abdominal Stoma

Table 1 presents the participants’ QoL results. The patients’ average QoL scores were 6.86 (SD = 0.84). Of these, 82.6% (*n* = 19) had a moderate QoL, and 17.4% (*n* = 4) had a high QoL. None of the participants rated the QoL as low. Among the different domains, spiritual well-being had the highest mean score (8.07; SD = 1.17), while physical well-being had the lowest mean score (6.29; SD = 0.76).

### Factors Affecting the QoL of Patients with Abdominal Stoma

Table 2 shows the results of data analysis on associations between sociodemographic characteristics, clinical factors, and QoL in patients with abdominal stoma. The result indicated that employment status (*P* = 0.028) and ability to care for a stoma (*P* = 0.039) were significantly associated with QoL. The mean QoL of employed patients was higher (7.58; SD = 0.69) than in unemployed or retired patients (6.66; SD = 0.78). Patients who cared for a stoma independently had a higher mean QoL (7.78; SD = 0.62) than patients who required help from others (6.72; SD = 0.79).

Additional model diagnostics showed that the model assumptions were fulfilled, with no significant interactions among the independent variables and no multicollinearity detected. Multiple linear regression analysis demonstrated a significant negative association between employment status and QoL (*b* = −0.94 [95% CI: −1.67, −0.22]; *P* = 0.014), and a significant positive association between a diagnosis of CRC and QoL (*b* = 0.92 [95% CI: 0.13, 1.71]; *P* = 0.025). Although the crude regression coefficient for stoma care (*b* = −1.059) suggested that dependence in stoma care was associated with significantly lower QoL scores compared to independence (*P* = 0.039), this variable was not included in the adjusted model, due to non-significance after adjustment. The final model accounted for 38.9% of the variance in QoL scores (*R*^2^ = 0.389), identifying employment status and CRC diagnosis as significant predictors of QoL (Table 3).

## Discussion

The present study assessed the QoL of patients living with abdominal stomas and examined the influence of sociodemographic and clinical factors on their QoL. Overall, the majority of participants reported a moderate QoL, while a smaller proportion experienced high QoL. These findings are comparable to stoma-related QoL outcomes reported in international studies (22–24). Previous research indicates that both living conditions and the healthcare environment significantly affect the QoL of people with stomas, with studies from developed countries generally reporting higher QoL. For example, a latent profile analysis involving 1,419 people with stomas in the United Kingdom found that 75.8% had either consistently good QoL or only mild QoL concerns (23). Similarly, a study conducted in New Zealand using three different QoL instruments among individuals with colostomy or ileostomy demonstrated consistently high QoL levels (25). In contrast, studies conducted in Vietnam, India, and Nepal reported lower to moderate QoL levels, potentially attributable to limited access to ostomy-related health services and disparities in supportive care (1, 13, 26).

This study’s findings were consistent with recent research showing that physical well-being is the most affected dimension of QoL, while spiritual well-being is the least affected (24). A study conducted among Chinese people with abdominal stomas in Beijing reported that daily routines and stoma management were among the most affected areas of life (12). This may partly explain the pronounced decline in physical well-being, particularly within the first three months after surgery (27). For many patients, practical concerns, including clothing limitations, changes in dressing style, and the need for continuous lifestyle adjustments, remain ongoing challenges that affect daily comfort and overall QoL (26, 28). Additionally, stoma location has also been shown to significantly affect patients’ QoL and their ability to adjust, with right-sided stomas among cancer survivors linked to postoperative weight gain, increased skin irritation, and lower satisfaction with appearance (16, 28). Nevertheless, evidence from a quasi-experimental study in Iran showed that physical well-being can improve over time when patients develop better adaptation and coping strategies for living with their stoma (29). Thus, early individualised stoma care education, incorporating a comprehensive assessment of physical challenges prior to discharge and regular evaluation of patients’ adjustment difficulties during follow-up, should be emphasised as essential nursing interventions to facilitate smoother adaptation and enhance long-term QoL outcomes.

Spirituality is a dynamic and evolving construct expressed through an individual’s beliefs, practices, and lived experiences that foster a sense of connection with something that promotes personal growth and leads to positive feelings and values (30). As spirituality and religiosity are closely interconnected and shaped by cultural context, individuals frequently rely on their religious beliefs and cultural backgrounds to interpret and find meaning in life events (30). In the present study, almost all participants identified as Muslims. The study setting adheres to the hospital’s Shariah-compliant policy, which ensures the availability of appropriate facilities and trained staff to support patients and visitors in fulfilling their religious obligations. Such an environment is likely to foster spiritual well-being and contribute positively to the overall QoL of patients with abdominal stomas. It is therefore plausible that participants’ levels of spiritual well-being remained high, as their religious practices were preserved during hospitalisation. The ability to pray and seek spiritual support may have strengthened their coping mechanisms and optimism in facing illness-related challenges.

Financial security emerged as a significant determinant of QoL among patients with abdominal stomas. Evidence from a cross-sectional study involving Colombian CRC patients indicates that satisfaction with income is closely related to QoL, primarily because adequate financial resources enable individuals to meet the ongoing demands of stoma care (31). However, another study suggests that difficulties in purchasing stoma bags may not stem solely from financial hardship. Rather, stoma-related complications such as leakage and peristomal skin irritation necessitate more frequent pouch replacement, thereby increasing overall expenditure (32). Stoma effluent contains proteolytic and lipolytic enzymes capable of damaging both protein- and lipid-based components of the peristomal skin’s epithelial barrier (33). Moreover, ileostomy output is thinner and richer in digestive enzymes, increasing the risk of peristomal skin irritation and injury compared with colostomies (34). In the present study, employed individuals tended to report better QoL, highlighting the importance of nurses carefully assessing patients at risk of financial strain. This involves identifying accessible support schemes and strengthening preventive measures to reduce stoma-related complications, as these complications not only impair QoL but also escalate the costs of stoma management and overall healthcare utilisation.

Patients with CRC in this study tended to report a moderate to higher perceived QoL. One plausible explanation is that individuals with CRC often benefit from more structured and comprehensive support systems, which facilitate the development of effective coping mechanisms and positively influence their appraisal of QoL. For instance, in Malaysia, the non-governmental organisation (NGO) National Cancer Council Malaysia assists in alleviating the financial burden of cancer-related care, including stoma care supplies, alongside psychosocial support from healthcare providers and trained volunteers. Such resources may buffer the psychological and physical stressors associated with cancer and stoma management. In contrast, stoma formation is also common in non-cancer conditions, particularly IBD, which is characterised by a relapsing-remitting course (35). Patients with non-cancer stomas may therefore have reduced access to comparable support services, placing them at greater risk of poorer QoL. These findings underscore the need for healthcare providers implementing systematic screening for psychosocial needs, financial challenges, and gaps in coping resources to ensure holistic and equitable care for all individuals living with a stoma.

Overall, the model of factors influencing QoL represented by employment status and having CRC accounted for 38.9% of the variance in QoL scores, suggesting a moderate level of explanatory power. This proportion of explained variance is clinically meaningful and provides valuable insights for strengthening stoma care practices, particularly given that QoL is influenced by complex and multidimensional factors. Nevertheless, the findings indicate that more than half of the variability in QoL remains unexplained. Future research should therefore incorporate additional determinants, such as social support, stoma-related complications, and self-efficacy, which may play an important role in shaping patients’ perception of their QoL.

This study has several limitations that warrant consideration. The cross-sectional design precluded the assessment of changes in QoL over time, and the relatively small sample drawn from a single, newly established hospital limits the generalisability of the findings. Although the study was conducted during the COVID-19 pandemic, it offers an early assessment of ostomy care outcomes and underscores the importance of comprehensive and systematically maintained patient records. A high dropout rate was anticipated due to cancer-related mortality and palliative care needs; therefore, caregivers assisted with data completion, when necessary, with appropriate measures taken to ensure that responses accurately reflected patients’ experiences.

## Conclusion

In this study, the QoL of patients with abdominal stomas was found to be moderate. Physical well-being was the most affected domain, whereas spiritual well-being was the least affected, likely reflecting the patients’ underlying clinical conditions and the supportive religious context of care. Higher QoL was significantly associated with a diagnosis of CRC and employment status, highlighting the potential role of financial stability and access to structured support systems. These findings suggest the importance of comprehensive, equitable care that addresses not only physical challenges but also psychosocial, spiritual, and financial needs, particularly for patients with non-cancer-related stomas who may have fewer support resources. Future research should consider larger cohort studies and multi-centre designs to provide a more comprehensive understanding of the complex factors influencing Malaysian patients’ perceptions and experiences of QoL.

## Figures and Tables

**Figure 1:**
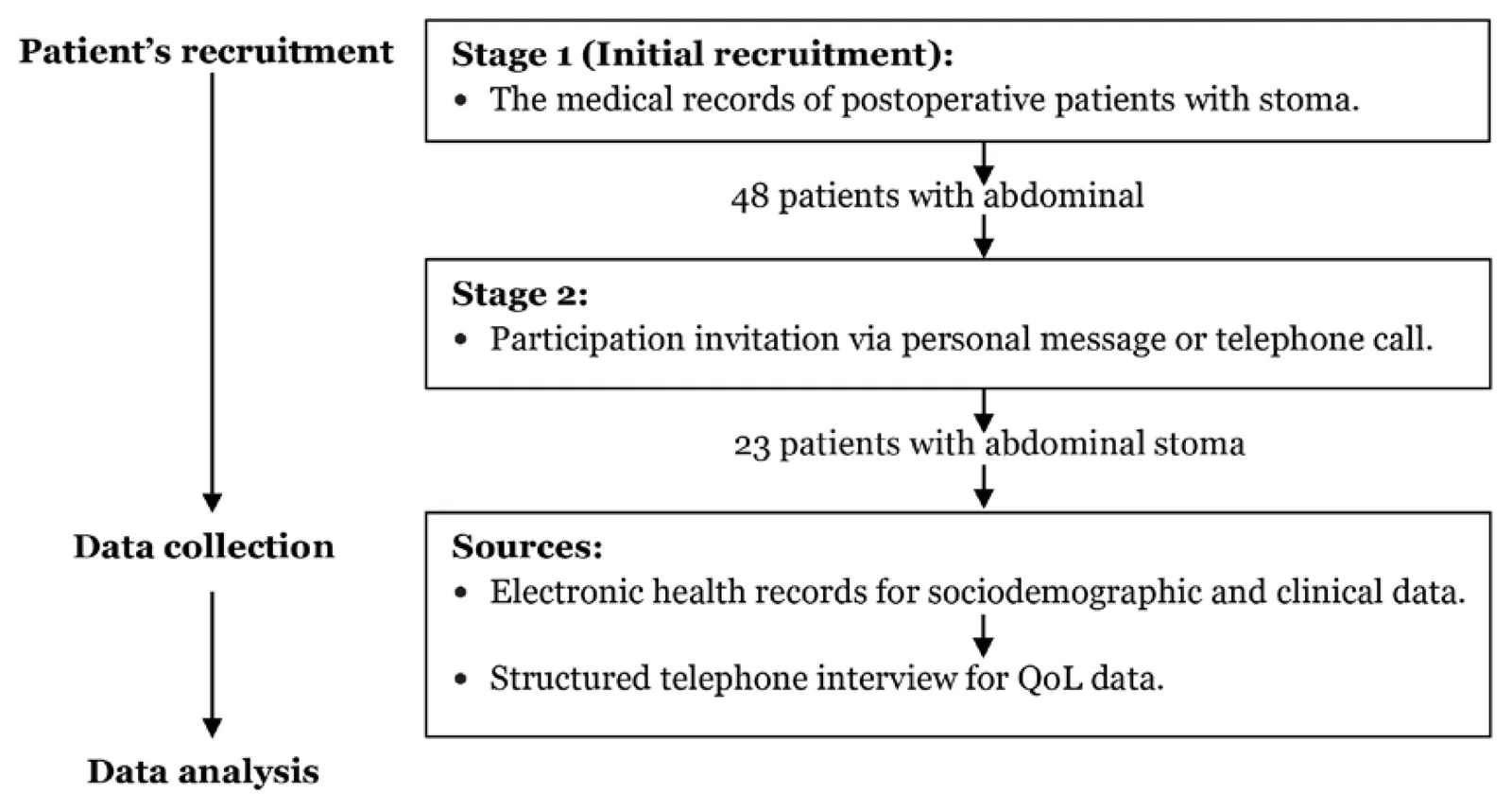
Recruitment of the participants and data collection

**Table 1:** QoL of participants with abdominal stoma (*n* = 23)

Ostomy QoL	Mean score	SD
Total score of QoL	6.86	0.84
Physical well-being	6.29	0.76
Psychological well-being	6.84	0.97
Social well-being	6.70	1.16
Spiritual well-being	8.07	1.17

**Table 2:** Factors associated with the QoL of participants living with abdominal stoma (*n* = 23)

Variable	Frequency (%)/Mean (SD)	Mean QoL (SD)	*b* (95% CI)	*t*-statistic	*P*-value
**Sociodemographic data**					

Age	58.70 (16.35)	6.86 (0.84)	−0.006 (−0.03, 0.02)	10.628	0.604

Gender					
Male^d^	16 (69.6)	6.92 (0.72)			
Female	7 (30.4)	6.74 (1.12)	−0.18 (−0.99, 0.63)	−0.463	0.648

Education					
Primary^d^	4 (17.4)	6.27 (0.71)			
Secondary	13 (56.5)	6.91 (0.82)	0.720 (−0.21, 1.65)^a^	1.613	0.122
Tertiary	6 (26.1)	7.15 (0.91)			

Marital status					
Married^d^	20 (87.0)	6.94 (0.86)			
Single/divorced/widowed	3 (13.0)	6.32 (0.52)	−0.626 (−1.70, 0.44)	−1.216	0.237

Employment status					
Employed^d^	5 (21.7)	7.58 (0.69)			
Unemployed/retired	18 (78.3)	6.66 (0.78)	−0.913 (−1.72, −0.11)^b^	−2.360	0.028

**Clinical data**					

Type of stoma					
Colostomy^d^	10 (43.5)	6.99 (0.83)			
Ileostomy	13 (56.5)	6.76 (0.87)	−0.231 (−0.98, 0.51)	−0.644	0.526

Stoma character					
Temporary^d^	19 (82.6)	6.93 (0.79)			
Permanent	4 (17.4)	6.56 (1.15)	−0.361 (−1.33, 0.61)	−0.774	0.447

CRC					
No^d^	4 (17.4)	6.13 (0.43)			
Yes	19 (82.6)	7.02 (0.83)	0.882 (−0.02, 1.78)	2.040	0.054

Pre-op site marking					
No/Not sure^d^	8 (34.8)	6.71 (0.98)			
Yes	15 (65.2)	6.94 (0.78)	0.230 (−0.55, 1.01)	0.617	0.544

Stoma duration					
≤ 3 months^d^	8 (34.8)	7.01 (0.85)			
> 3 but < 12 months	9 (39.1)	7.18 (0.81)	−0.229 (−1.01, 0.55)^c^	−0.612	0.547
12 months or above	6 (26.1)	6.19 (0.54)			

Stoma complication					
No^d^	17 (73.9)	6.81 (0.82)			
Yes	6 (26.1)	7.02 (0.96)	0.212 (−0.63, 1.06)	0.523 0.607	

Stoma care					
Independence^d^	3 (13.0)	7.78 (0.62)			
Dependence	20 (87.0)	6.72 (0.79)	−1.059 (−2.06, −0.06)	−2.205	0.039

a Data were categorised into two groups: i) primary and ii) secondary and tertiary;

b Data were categorised into two groups: i) unemployed and ii) employed or retired;

c Data were categorised into two groups: i) ≤ 3 months and ii) > 3 months;

d Reference group; a *P*-value < 0.05 is significant;

CI = confidence interval; SD = standard deviation

**Table 3:** Multiple linear regression model of factors associated with the QoL of participants living with abdominal stoma (*n* = 23)

Variable	Simple linear regression			Multiple linear regression		
	
	*b*^a^ (95% CI)	*t*-statistic	*P*-value	*b*^b^ (95% CI)	*t*-statistic	*P*-value
Employment status						
Employed^c^						
Unemployed/retired	−0.913 (−1.72, −0.11)	−2.360	0.028	−0.943 (−1.67, −0.22)	−2.706	0.014

CRC						
No^c^						
Yes	0.882 (−0.02, 1.78)	2.040	0.054	0.919 (0.13, 1.71)	2.424	0.025

Stoma care						
Independence^c^						
Dependence	−1.059 (−2.06, −0.06)	−2.205	0.039	–	–	–

a Crude regression coefficient;

b adjusted regression coefficient;

c reference group;

Forward multiple linear regression method applied; A *P*-value < 0.05 is significant
